# Engineered exosomes for targeted co-delivery of miR-21 inhibitor and chemotherapeutics to reverse drug resistance in colon cancer

**DOI:** 10.1186/s12951-019-0563-2

**Published:** 2020-01-09

**Authors:** Gaofeng Liang, Yanliang Zhu, Doulathunnisa Jaffar Ali, Tian Tian, Huantian Xu, Ke Si, Bo Sun, Baoan Chen, Zhongdang Xiao

**Affiliations:** 1 Department of Hematology, Zhongda Hospital, Medical School of Southeast University, Nanjing, 210009 Jiangsu China; 2 Medical College, Henan University of Science and Technology, Luoyang, 471023 Henan China; 3 State Key Laboratory of Bioelectronics, School of Biological Science and Medical Engineering, Southeast University, Nanjing, 210096 Jiangsu China; 4 Department of Neurobiology, Key Laboratory of Human Functional Genomics of Jiangsu, Nanjing Medical University, Nanjing, 211166 Jiangsu China

**Keywords:** Exosomes, Delivery system, miR-21 inhibitor, 5-FU, Drug resistance

## Abstract

**Background:**

5-Fluorouracil (5-FU) has been commonly prescribed for patients with colorectal cancer (CRC), but resistance to 5-FU is one of the main reasons for failure in CRC. Recently, microRNAs (miRNAs) have been established as a means of reversing the dilemma by regulating signaling pathways involved in initiation and progression of CRC. However, how to safely and effectively deliver miRNA to target cells becomes a main challenge.

**Results:**

In this study, Engineered exosomes were exploited to simultaneously deliver an anticancer drug 5-FU and miR-21 inhibitor oligonucleotide (miR-21i) to Her2 expressing cancer cells. Purified engineered exosomes from the donor cells loaded with 5-FU and miR-21i via electroporation to introduce into 5-FU-resistant colorectal cancer cell line HCT-116^5FR^. Furthermore, systematic administration of 5-FU and miR-21i loaded exosomes in tumor bearing mice indicated a significantly anti-tumor effect. The results showed that the engineered exosome-based 5-FU and miR-21i co-delivery system could efficiently facilitate cellular uptake and significantly down-regulate miR-21 expression in 5-FU resistant HCT-116^5FR^ cell lines. Consequently, the down-regulation of miR-21 induced cell cycle arrest, reduced tumor proliferation, increased apoptosis and rescued PTEN and hMSH2 expressions, regulatory targets of miR-21. Of particular importance was the significant reduction in tumor growth in a mouse model of colon cancer with systematic administration of the targeting miR-21i. More excitedly, the combinational delivery of miR-21i and 5-FU with the engineered exosomes effectively reverse drug resistance and significantly enhanced the cytotoxicity in 5-FU-resistant colon cancer cells, compared with the single treatment with either miR-21i or 5-FU.

**Conclusion:**

The strategy for co-delivering the functional small RNA and anticancer drug by exosomes foreshadows a potential approach to reverse the drug resistance in CRC and thus to enhance the efficacy of the cancer treatment.

## Introduction

Colorectal carcinoma (CRC) is the third most lethal cancer worldwide, and shows a higher morbidity because of its aggressive behavior, poor prognosis, and lack of targeted treatments [1]. 5-FU based chemotherapy plays an important role in treatment of CRC. However, the therapeutic effect is severely weakened by the multidrug resistance (MDR) caused by a successful long-term administration of 5-FU. Many mechanisms such as gene mutation, DNA methylation and histone modification are involved in the resistance of cancer cells to chemotherapeutic agents [2-4]. Recently, many efforts have been exerted in analyzing the role of miRNAs in the development of drug resistance in a variety of cancers, which have shown that chemoresistant cancer cells and their parental chemosensitive ones have distinct miRNA expression patterns, and the molecular targets and mechanisms of chemoresistance also have been elucidated [5, 6]. Valeriet al further revealed that miR-21 induced resistance to 5-FU by down-regulating human DNA MutS homolog 2 (hMSH2) in colorectal cancer [7]. Furthermore, it has shown that restoration of the dysregulated miRNAs can effectively overcome drug resistance [8-10]. Therefore, we speculate that the co-delivery of MDR-reversing miRNA and chemotherapeutics will be a promising way to overcome MDR in cancer chemotherapy [11, 12]. However, a safe and efficient targeted delivery system is pivotal for CRC therapy.

Exosomes are small (40–120 nm) membrane vesicles of endocytic origin and are released into the extracellular environment during the fusion of multivesicular bodies (MVB) with the plasma membrane [13]. The presence of mRNAs, miRNAs, and proteins are examined in the exosomes derived from various cells to define a potential mechanism by which exosomes may mediate cell–cell communication in vivo [14-18]. The characteristics of exosomes that facilitate an efficient delivery of biological drugs include their capacity to cross the intact biological barriers (e.g. blood–brain barrier) and deliver functional RNAs and small molecule drugs into cells, as well as their stability in blood [19-21]. Moreover, exosomes can gain the tumor targeting ability easily via molecular biology methods as they are naturally derived from cells [22]. Inspired by this, increasing studies focus on exploiting these features to engineer natural exosomes for drug or functional nucleic acid delivery to specific disease tissues [23, 24]. However, co-delivery of functional RNAs and chemotherapy drugs using exosomes has not been analyzed in detail, thus the biological parameters and therapeutic potential of exosomes for multicomponent delivery still need further investigation.

**Scheme 1:**
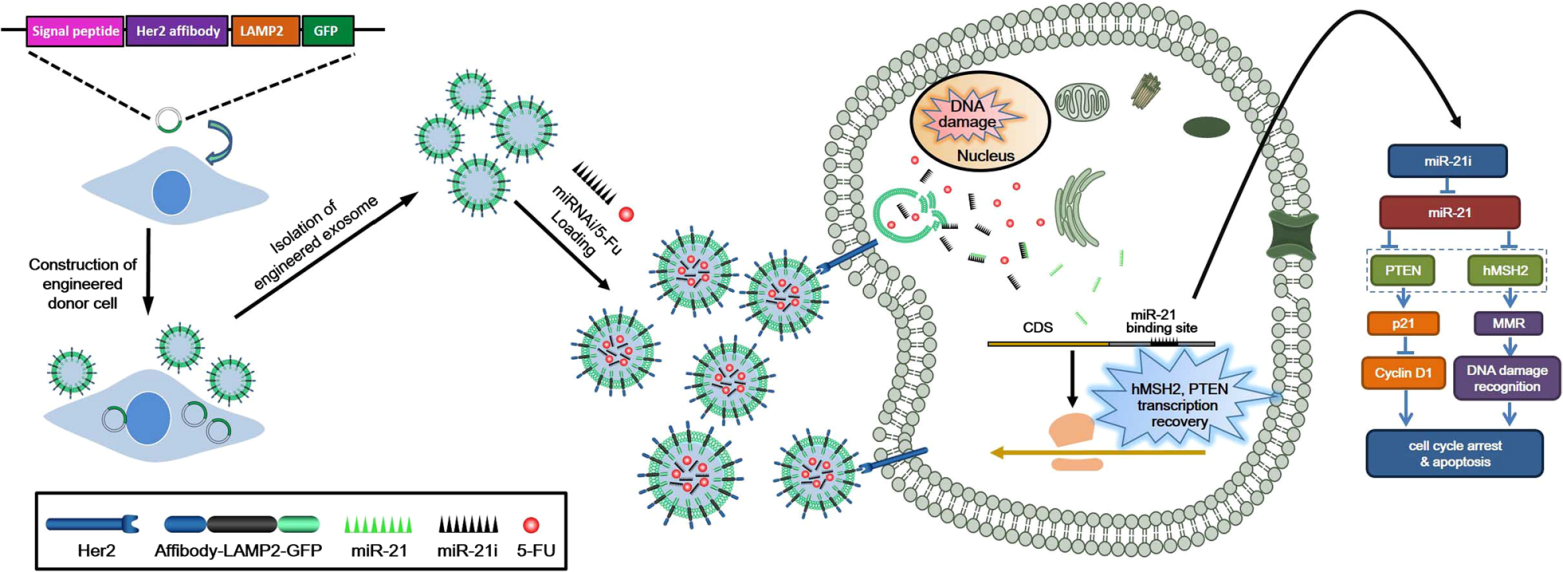
Engineered exosomes based nanocarrier for 5-FU and miR-21i simultaneously deliver to HCT-116^5FR^ human colon cancer cells for enhancing chemotherapy efficacy

Herein, we developed a strategy to produce target-specific exosomes to co-deliver miR-21 inhibitor (miR-21i) and chemotherapeutic drugs into the 5-FU-resistant HCT-116(HCT-116^5FR^) cells, a colorectal cancer cell line in which miR-21 has been reported to be highly expressed. To confer targeting capability to exosomes, Her2, a widely expressed membrane protein that is involved in tumor progression and suppression, as a specific tumor-homing polypeptide to achieve cancer cells targeting. Firstly, Her2 was fused with the LAMP2 to efficiently target colon cancer cells HCT-116^5FR^, a well-established drug resistant cancer cell. The Her2-LAMP2 fusion protein was shown to be expressed on the surface of exosomes where it facilitated the targeted cellular uptake through by EGFR receptor-mediated endocytosis in HCT-116 cells. Target-specific exosomes were generated at a high yield by engineered 293T cells. 5-FU and miR-21i packaging into engineered exosome (target-Her2-LAMP2-GFP, THLG-Exo) was achieved by mixing an appropriate concentration of 5-FU with Exo by Electroporation. Then miR-21i was co-incubated with THLG-Exo/5FU to form a co-delivery system (THLG-EXO/5-FU/miR-21i, Scheme 1). These engineered exosomes were evaluated in terms of their targeting and therapeutic effects, both in vitro and in vivo.

## Results

### Isolation and characterization of exosomes

**Fig. 1:**
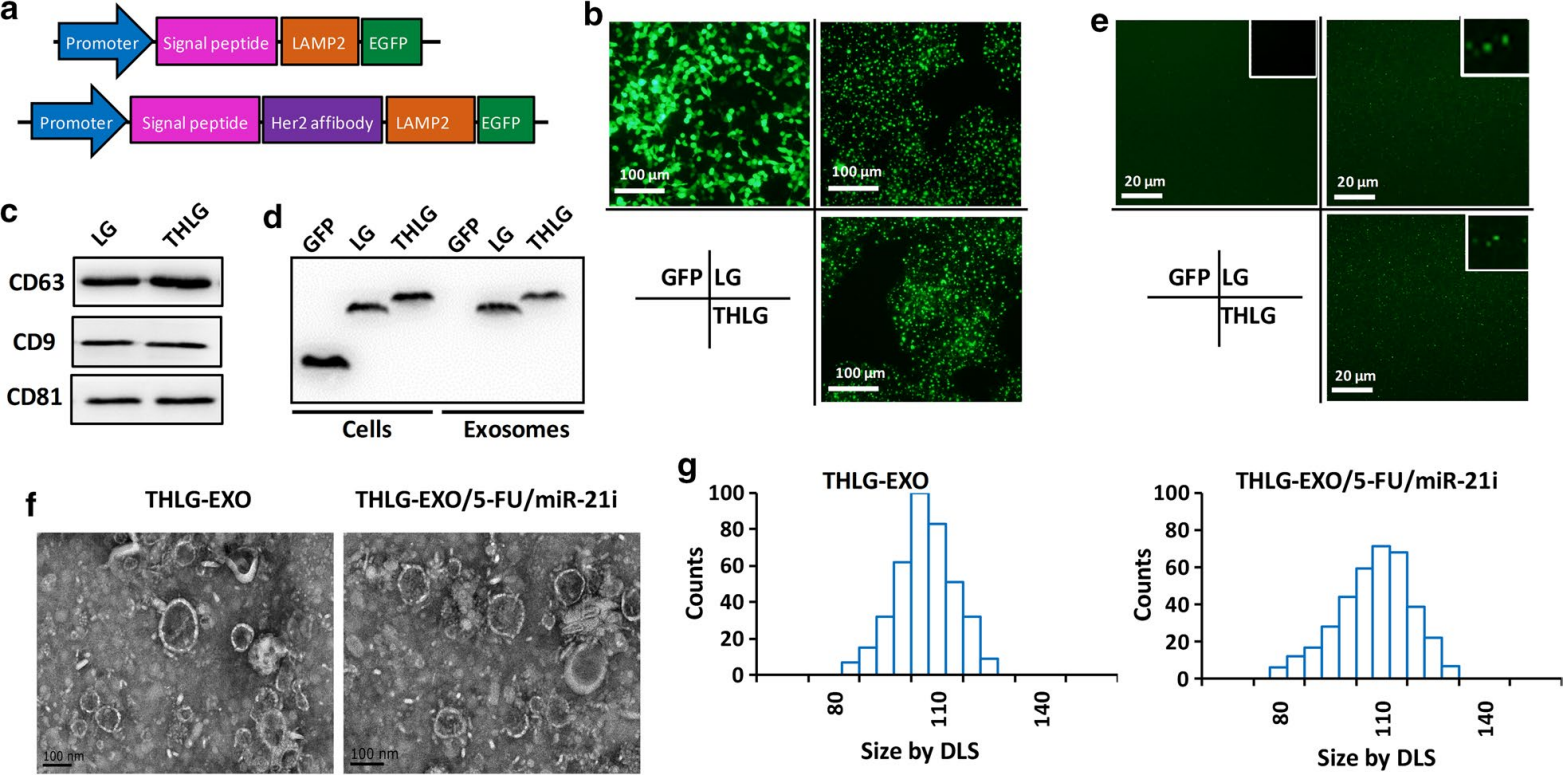
Isolation and molecular characterization of engineered exosomes. **a** Schematics of DNA constructs used for the production of fusion proteins LG (upper) and THLG (lower). **b** Fluorescence images of the GFP, LG and THLG in 293T cells ( ×400). **c** Biomarkers of exosomes were determined by western blot analysis of exosomes. **d **Western blot analysis of GFP in exosomes obtained from supernatants of GFP-293T cells, LG-293T cells and THLG-293T cells. **e** Confocal microscopy of exosomes isolated from 0.22 filtered conditioned medium of GFP-293T cells, LG-293T cells and THLG-293T cells ( ×1000). **f** Transmission electron micrograph of THLG-EXO and THLG-EXO/5-FU/miR-21i.** g** Size distribution of THLG-EXO and THLG-EXO/5-FU/miR-21i measured by DLS

To confer targeting capabilities, we fused Her2-binding affibody to the extra-exosomal N terminus of human Lamp2, a protein found abundantly in exosomal membranes according to the previous research [25], and then cloned into pLVX-GFP-N1, so the final fusion protein consist of Her2-binding affibody, LAMP2 and GFP, named THLG. Another fusion protein containing LAMP2 and GFP without Her2-binding affibody was also constructed and named LG as control (Fig. 1a). HEK293T cells were then stably transduced with a lentivirus vector encoding either THLG or LG. Fluorescence microscope of THLG-293T or LG-293T cells showed that THLG and LG chimera proteins were located in the granule membranes as well as in the plasma membranes (Fig. 1b).The exosomes were purified from the culture supernatants of THLG-293T or LG-293T cells by ultracentrifugation (henceforth referred to as THLG-EXO and LG-EXO respectively), and the markers of exosomes, such as CD63, CD9 and CD81 were determined by western (Fig. 1c). To validate whether the LAMP2 fusion protein was incorporated into the exosomes from parent cells, the expression of fusion protein in parent cells and derived exosomes was detected by western blot analysis with anti-GFP antibody. As shown in Fig. 1c, western blot analysis showed that the molecular weight of the fusion proteins (LG) were around 80 kDa, and THLG fusion proteins was slightly higher than LG, which was in good agreement with the sum of the molecular sizes of glycosylated LAMP2 and GFP. Furthermore, the results also revealed that LG and THLG were strongly expressed in HEK293T cells and were incorporated into the exosomes. By contrast, the exosomes derived from HEK293T cell transfected with GFP had undetectable levels of GFP, suggesting that the GFP in cytoplasm may not integrated into the exosomes (Fig. 1d). In addition, the above results proved the fusion protein comprising GFP existed in the exosomes, furthermore THLG-EXO or LG-EXO were tested which could be observed by laser scanning confocal microscope (Nikon, Japan). This result further indicated that the fusion protein was successfully incorporated into the exosomes (Fig. 1e).

Then the morphology and size of the exosomes were assessed using TEM and dynamic laser scatter (DLS). Specifically, THLG-EXO had typical saucer-like bilayer membrane structures, with diameters of 60–130 nm (Fig. 1f). DLS showed that the exosomes had a narrow size distribution, with a mean diameter of 97 nm, while 110 nm after 5-Fu/miR-21i loaded into THLG-EXO (Fig. 1g).

## Preparation of exosomal formulations of miR-21i and 5-FU

Next the feasibility of THLG-EXO to be used as a carrier to co-deliver 5-FU and miR-21i was evaluated. Several previously published results have shown that chemotherapeutic agents and foreign short RNAs can be effectively introduced into EVs using electroporation [6, 10, 22]. In this study miR-21i and 5-FU were loaded into exosomes by electroporation. To get the optimum parameters of electroporation, the effect of different varied voltages and pulse lengths on the transformation efficiency were tested. The results showed that the maximum loading efficiency was obtained when the time constant was 10 ms and the voltage was 1000 V. After the electroporation, the volume of THLG-EXO/5FU/miR-21i showed some modest differences in the topography of the miR-21i and 5FU-loaded exosomes, including slightly larger average diameter of 110 ± 11.3 nm (Fig. 1f, g) and surface potential of − 11 ± 2.7 mV. While TEM photographs of THLG-EXO revealed an average diameter of 97 ± 6.2 nm and surface potential of − 8 ± 2.4 mV. Regarding these variations, we speculate that it is a consequence of electroporating miR-21i and 5-FU in exosomes, as all other procedures used to produce exosomes were identical. Under the optimum electroporation conditions, the results of HPLC and qRT-PCR indicated that the 5-FU and miR-21i loading capacity (LC) of exosomes were approximately 3.1% and 0.5%, respectively.

## In vitro targeting of THLG-EXO

**Fig. 2:**
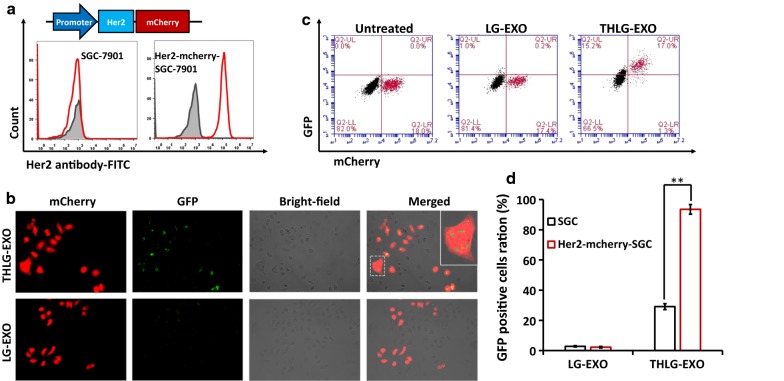
The cellular tropism of THLG-EXO in vitro. **a** Schematics of DNA constructs used for the production of fusion proteins Her2-mCherry (upper panel); flow cytometric analyses of Her2 from SGC-7901 WT cells and Her2-mcherry-SGC-7901 cells with a FITC labeled anti-Her2 monoclonal antibody (lower panel). **b** Confocal microscopy images of cellular uptake of THLG-EXO or LG-EXO after 3 h incubation with SGC-7901 WT cells and Her2-mcherry-SGC-7901 cells co-culture model. red shows Her2-mcherry-SGC-7901 cells. Green represents THLG-EXO or LG-EXO. Right panel shows enlarged graphs with frame indicating internalized exosomes in cells (×400). **c** Flow cytometry analysis of co-culture cells after incubation with THLG-EXO or LG-EXO (upper panel); **d** quantification of exosomes internalization based on flow cytometry analysis (lower panel). Data are expressed as mean ± SD. n = 5; **p < 0.01

In order to analyze the potential targeting ability of THLG-EXO in vitro, a Her2-negative SGC-7901 WT cells and Her2-positive Her2-mcherry-SGC-7901 cells co-culture model were established and used for the evaluation (Fig. 2a). Fluorescence microscope results showed that THLG-EXO entered more efficiently into Her2-mcherry-SGC-7901 cells as compared with SGC-7901 WT cells after the co-culture model was incubated with THLG-EXO for 3 h. By contrast, LG-EXO did not show such a specific localization towards Her2-mcherry-SGC-7901 cells and were randomly distributed in the co-culture model (Fig. 2b). Furthermore, cellular tropism of THLG-EXO was quantitatively analyzed using flow cytometry. As shown in Fig. 2c, in the co-culture model incubated with THLG-EXO for 3 h, the percent of GFP-positive Her2-mcherry-SGC-7901 cells were increased up to 92.9% compared with 18.6% of SGC-7901 cells with a significant difference (p < 0.01). By contrast, the percent of GFP-positive Her2-mcherry-SGC-7901 cells were increased only up to 1.4% compared with 1.2% of SGC-7901 cells after incubation with LG-EXO for 3 h. These results suggested that the THLG-EXO possessing targeting Her2 protein moieties on their surface could be recognized by the extracellular region of Her2 on the outer cellular membrane of Her2-mcherry-SGC-7901 cells which could evidently facilitate the uptake of THLG-EXO by Her2-mcherry-SGC-7901 cells, indicating that employing T-Her2 as the ligand for Her2 dramatically enhanced the binding ability of exosomes to target cells.

## Anti-tumor effect of THLG-EXO/5-FU/miR-21i in vitro

**Fig. 3:**
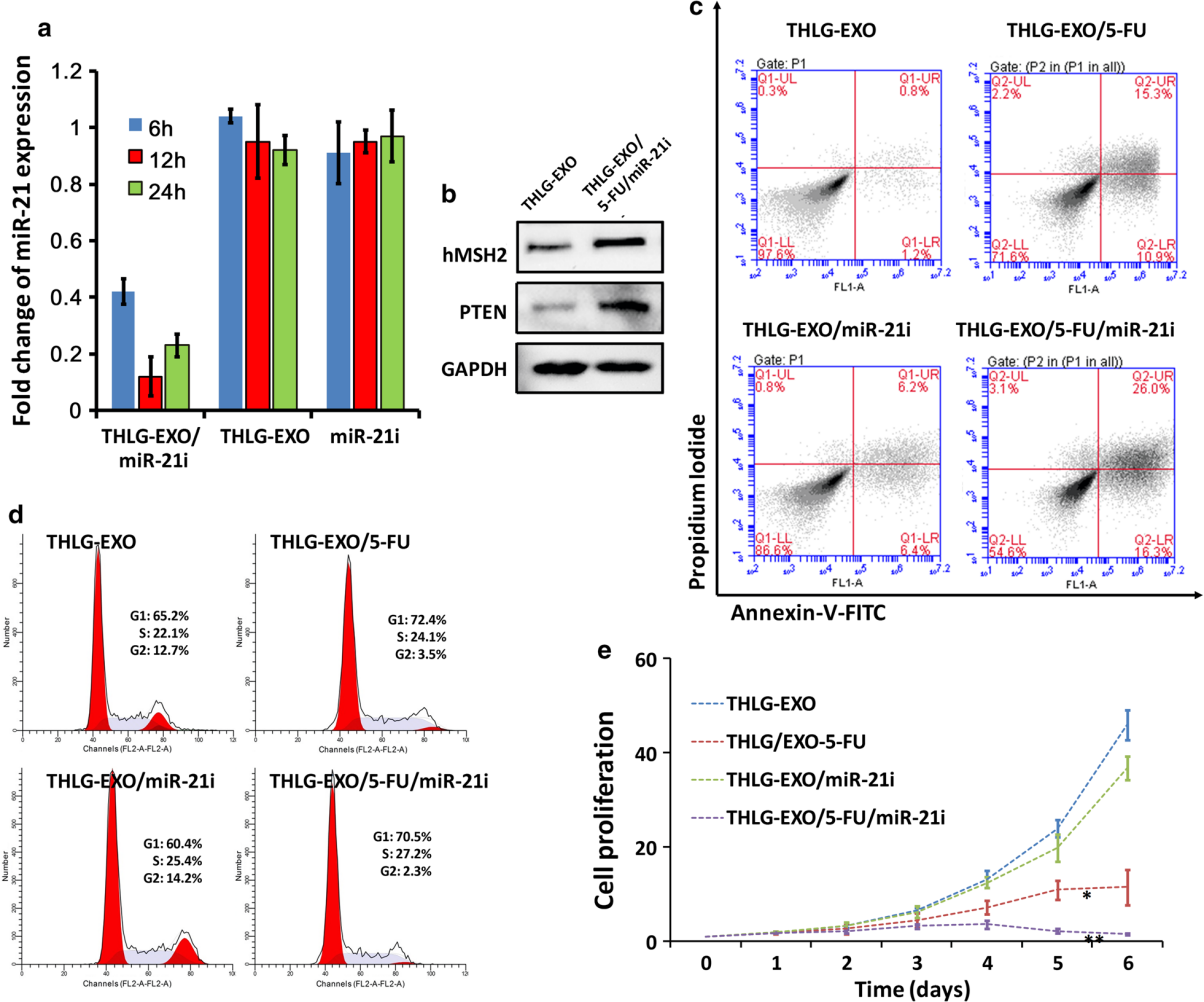
The results of HCT-116^5FR^ cells treated with THLG-EXO/5-FU/miR-21i in vitro. **a** Real-time fluorescent quantitative PCR analysis of relative expression change of miR-21 level in HCT-116^5FR^ cells treated with THLG-EXO/5-FU/miR-21i, THLG-EXO or miR-21i. Data were expressed as mean ± SD. n = 3. **b** Western blot analysis of hMSH2 and PTEN protein levels of HCT-116^5FR^ cells treated with THLG-EXO/5-FU/miR-21i. **c **Flow cytometric analysis of AnnexinV-FITC/PI stained HCT-116^5FR^ cells treated with THLG-EXO, THLG-EXO/miR-21i, THLG-EXO/5-FU or THLG/5-FU/miR-21i. **d ** Cell cycle profiles of HCT-116^5FR^ cells after treatment as described in **c**; **e** the proliferation of HCT-116^5FR^ cells after treatment as described in **c**. Data were expressed as means ± SD. n = 5;*: significantly different from other groups (p < 0.05), **: extremely significantly different from other groups (p < 0.01)

The above results indicated that the engineered exosomes were efficiently taken up by recipient cells. Followed by this, the biological effects induced by miR-21i and 5-FU encapsulated THLG-EXO and the synergetic cytotoxicity of miR-21i and 5-FU in 5-FU-resistant colon cancer cell lines were investigated. A 5-FU-resistant derivative of the HCT-116 human colon cancer cell line (HCT-116^5FR^) was generated by serial passage of these cells in the presence of increasing 5-FU concentrations. At first, the capability of THLG-EXO/5-FU/miR-21i for efficiently driving the oligonucleotide into targeted cells was investigated. The results of previous research had exhibited that the apparent expression levels of miRNA detected by qRT-PCR would be reduced by anti-miRNA, as the stable formation of base-pairing between mature miRNA and anti-miRNA molecules prevented the binding of the miRNA-specific looped RT-primer and subsequently inhibited the reverse transcription process. As illustrated in Fig. 3a, the endogenous miR-21 in HCT-116^5FR^ reduced dramatically as early as 6 h after incubation with THLG-EXO/miR-21i, whereas it was not the case in the incubation with free miR-21i, indicated no oligonucleotide internalization in HCT-116^5FR^ cells incubated with free miR-21i, as expected. To further confirm the biological function induced by miR-21i of THLG-EXO/5-FU/miR-21i in HCT-116^5FR^ cells, we evaluated the protein expression levels of the downstream targets of miR-21, such as hMSH2 and PTEN, the validated targets of miR-21, which were responsible for the miR-21 induced drug resistance [7, 26]. As anticipated, an increase in the protein expression level of hMSH2 and PTEN was detected in HCT-116^5FR^ cells treated with THLG-EXO/5-FU/miR-21i compared with THLG-EXO-treated cells (Fig. 3b). These results suggested that THLG-EXO/5-FU/miR-21i could efficiently deliver miR-21i into the cytoplasm, and specifically silence the biological function of miR-21 in the recipient cells.

Next, to evaluate the synergistic anti-tumor efficacy of THLG-EXO-mediated co-delivery of 5-FU and miR-21i, the apoptosis, cell cycle and proliferation of HCT-116^5FR^ cells were investigated at a 5-FU concentration of 5 μg/mL. As shown in Fig. 3c, the obtained FCM profiles reflected the cellular apoptosis after the treatments. The proportion of apoptotic cells (%) was comprised of late apoptotic (district of the upper right) and early apoptotic cells (district of the bottom right). The apoptotic proportion of HCT-116^5FR^ cells with either miR-21i or 5-FU loaded THLG-EXO, reached 12.6% and 26.2% respectively. Obviously, the single delivery of miR-21i did not achieve perfect therapy efficacy, in comparison with that of treated mock-THLG-EXO (apoptosis rate ~ 2.0%), which indicated a lack of powerful therapeutic function for single miR-21i. By comparison, THLG-EXO mediated co-delivery of 5-FU together with miR-21i led to a substantial enhancement of apoptosis (apoptosis rate ~ 42.3%) than that of the cells treated with THLG-EXO/5-FU at same 5-FU concentration(apoptosis rate ~ 26.2%), which demonstrated an enhanced synergistic effect on apoptosis induction in the target cells.

Subsequently, the cell cycle was evaluated by PI, and the results showed that THLG-EXO/5-FU/miR-21i caused considerably S-phase arrest (27.2% and 22.1%) in HCT-116^5FR^ cells than THLG-EXO, while THLG-EXO/miR-21i did not obviously affect HCT-116^5FR^ cell cycle distribution (Fig. 3d). It should be noted that THLG-EXO/5-FU/miR-21i also affects G1 cell cycle to a certain extent (70.5% and 65.2%) compared with THLG-EXO in HCT-116^5FR^ cells, but the effect was not as obvious as its effect on the S phase due to the relatively large proportion of G1 cells. As expected, the cell cycle assay presented a good correlation with the previous apoptosis data. In addition, we also analyzed the ability of THLG-EXO/miR-21i, THLG-EXO/5-FU and THLG-EXO/5-FU/miR-21i to inhibit the cancer cell proliferation. The results showed that all three formulations could inhibit cell proliferation, but as time went on, there was an obvious difference among cell proliferation inhibition in different formulations. As shown in Fig. 3e, cell proliferation was inhibited for ~ 12%, 43%, and 82% after incubation with THLG-EXO/miR-21i, THLG-EXO/5-FU and THLG-EXO/5-FU/miR-21i for 6 day, respectively. This result indicated that even though THLG-EXO/5-FU exhibit considerable growth inhibitory effect, it is worth noting when compared with THLG-EXO/5-FU/miR-21i which showed more intense effects on the cell proliferation inhibition, with almost completely inhibiting the cell proliferation. While the cell treated with THLG-EXO/miR-21i showed a slight decrease in the proliferation. This observation was consistent with our findings on the cell cycle arrest and apoptosis results. Thus the results suggested that miR-21i can increase the sensitivity of 5-FU on HCT-116^5FR^ cells, and the combination therapy with 5-FU and miR-21i can yield significantly enhanced antitumor efficacy compared with the single-agent treatment.

## In vivo distribution of THLG-EXO

**Fig. 4:**
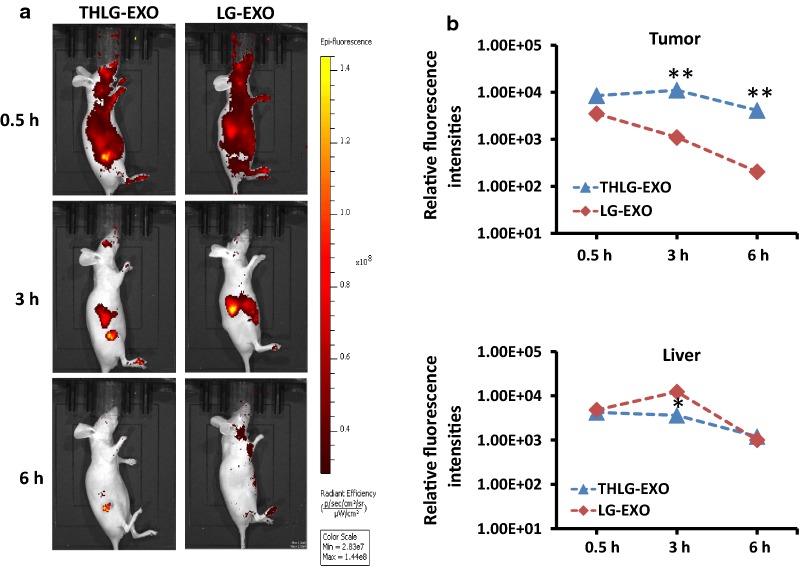
Tumor targeting ability of THLG-EXO in a nude mouse xenograft model using HCT-116^5FR^ cells. **a** In vivo fluorescent images of HCT-116^5FR^-bearing nude mice after intravenous injection of DiR-labeled THLG-EXO (left panel) or LG-EXO (right panel). **b** Quantitative analysis showed the relative fluorescence intensities in the tumor region (upper panel) and the liver (lower panel) following administration of THLG-EXO or LG-EXO. Data were expressed as means ± SD. n = 5; *P < 0.05, **P < 0.01 versus the LG-EXO group at the same time point

THLG-EXO mediated co-delivery of miR-21i and 5-FU to the Her2-positive cancer cells had shown an ideal delivery effect in vitro. Subsequently, their delivery efficiency in vivo was evaluated using in vivo imaging system (Xenogen, Alameda, CA, USA). To demonstrate the tumor targeting ability in vivo, HCT-116^5FR^ cells were grown as subcutaneous xenografts in female BALB/c nude mice. Later, THLG-EXO and LG-EXO were labeled with DiR dye and injected via tail vein into the nude mice bearing approximately 0.2 cm^3^ tumors. The biodistribution of injected exosomes were monitored at different time points. Representative results shown in Fig. 4, at the earliest time point after injection (30 min), THLG-EXO and LG-EXO were rapidly distributed all over the body, with the difference that a large proportion of THLG-EXO distributed in the tumor, which was indicative of the fast accumulation within the tumor area, whereas LG-EXO was located mainly in liver, suggesting that LG-EXO uptake and retention took place primarily in the liver and other metabolic organs, with little accumulation in the tumor. Moreover, as time went on, striking differences were found among these two groups. A relatively intense fluorescence signal exclusively in the tumor area was detected after 3 h of injection of THLG-EXO with other parts of the body gradually decreased. Moreover, the relatively intense fluorescence signal still could be detected in the foot and neck after 6 h of injection of the LG-EXO. By contrast, no signal was detected at these sites in the THLG-EXO group, while the fluorescence signal only could be detected in tumor site, indicating a rapid blood clearance by reticuloendothelial system. Taken together, these data suggested that THLG-EXO could be served as an effective drug delivery carrier for targeting Her2-expressing tumor.

## Antitumor effect of THLG-EXO/5-FU/miR-21i in vivo

**Fig. 5:**
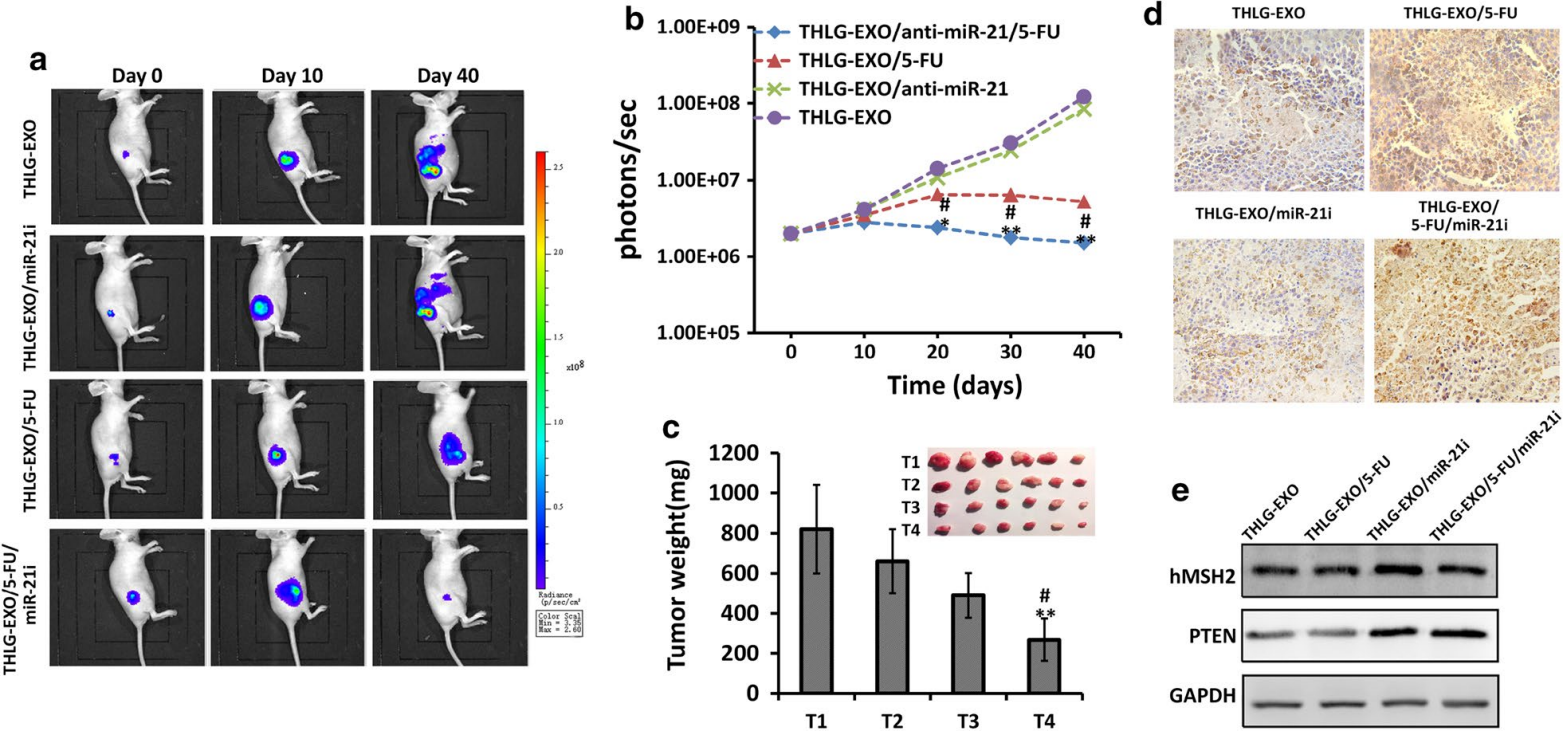
Antitumor activity of THLG-EXO/5-FU/miR-21i in nude mice xenograft using HCT-116^5FR^ cells. **a** Representative bioluminescent images of tumor growth in nude mice treated with THLG-EXO, THLG-EXO/miR-21i, THLG-EXO/5-FU or THLG-EXO/5-FU/miR-21i at different time point. Signals were adjusted to the same color scale for the entire time course. **b **The mean bioluminescence intensity (BLI) for each mouse/group, line chart represented quantification of the relative bioluminescent intensity in tumor site. **c** Tumor weights of different treated groups. T1, T2, T3 and T4 represented the treatment of THLG-EXO, THLG-EXO/miR-21i, THLG-EXO/5-FU, THLG-EXO/5-FU/miR-21i, respectively. **d** TUNEL assessments of tumor tissues treated with THLG-EXO, THLG-EXO/miR-21i, THLG-EXO/5-FU, THLG-EXO/5-FU/miR-21i, respectively. **e** Western blot of hMSH2 and PTEN in the tissue of tumor region were performed for 24 h after administration. Data were expressed as means ± SD. n = 6; *P < 0.05, **P < 0.01 versus the THLG-EXO groups or THLG-EXO/miR-21i group, and ^#^P < 0.05 versus the THLG-EXO/5-FU groups

Having shown the capacity of THLG-EXO for the efficient targeted delivery of drug to the tumor cells and the synergy of miRNA modulation and treatment with 5-FU in vitro, experiments were performed to assess whether this multimodal approach could result in an enhanced anti-tumor effect in vivo. Female nude mice bearing HCT-116^5FR^-Luc (luciferase expressing) induced tumors were randomly divided into four groups and administered with various formulations including THLG-EXO/5-FU/miR-21i, THLG-EXO/5-FU, THLG-EXO/miR-21i, and the group that treated with empty THLG-EXO was also established as control. Firstly, the impact of different formulations of exosomes on tumor growth was assessed for long time period. Due to the tumor bioluminescence in mice could be linearly correlated with the tumor volume [27, 28]. We estimated the tumor size by recording the bioluminescent intensity of the tumor sites. As expected, the best performance was achieved in the group received THLG-EXO/5-FU/miR-21i formulation, where the tumor volume shrank remarkably with time dependently. Representative results shown in Fig. 5a, the other treatments still resulted in the tumor expansion with different degrees, especially in the group of THLG-EXO, the metastasis of malignant tumor was even obvious. Furthermore, the mean bioluminescence intensity (BLI) for each mouse/group was quantified by measure the relative bioluminescent intensity (Fig. 5b). At the end of the investigation, the mice were sacrificed and the tumor was stripped out and weighed. As shown in Fig. 5c, obviously, compared with those groups injected with THLG-EXO and THLG-EXO/5-FU, mice injected with THLG-EXO/5-FU/miR-21i showed a significantly inhibition of tumor growth and decreased tumor weights.

To further evaluate apoptosis induced by different targeted exosomes formulations in the tumor, cellular apoptosis was analyzed using the TdT dUTP nick end-labeling (TUNEL) staining. As shown in the Fig. 5d, the number of TUNEL positive cells significantly increased after the intravenous injections of THLG-EXO/5-FU or THLG-EXO/5-FU/miR-21i, especially in the group of THLG-EXO/5-FU/miR-21i. Intravenous injections of THLG-EXO/miR-21i showed a slight increase in TUNEL-positive cells, suggesting that using miR-21i alone had little effect on cell apoptosis. However, combined with 5-FU, engineered exosomes including miR-21i could achieve a very significant anti-tumor effect. Subsequently, the gene regulating activity of miR-21i delivered by the exosomes was determined by western blot analysis. The results of Fig. 5e showed that the protein expression of hMSH2 and PTEN in the tumor tissue was rescued by the THLG-EXO/miR-21i and THLG-EXO/5-FU/miR-21i, while the solely delivered 5-FU by THLG-EXO had almost no noticeable effect on the expression of hMSH2 and PTEN. These in vivo results presented an upright correlation with the in vitro assays in the cells, and powerfully demonstrated the powerful synergism by virtue of THLG-EXO mediated co-delivery of miR-21i and 5-FU.

## In vivo safety evaluation

**Fig. 6:**
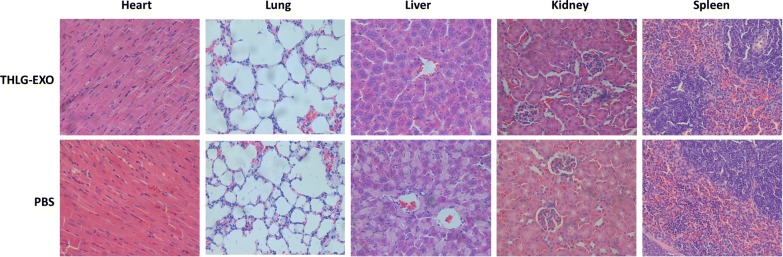
The systematic toxicity assessment of THLG-EXO. Histopathological analysis of heart, lung, liver, kidney and spleen sections stained with hematoxylin and eosin of BALB/c mice post-intravenous injection of 20 mg/kg THLG-EXO or PBS for 7 days (one dose every other day). Images were obtained under Nikon Ti microscope using a  × 40 objective

In addition to treatment efficacy, toxicity is another critical parameter of an excellent delivery vehicle for further use. For safety purpose, we evaluated the systematic toxicity of THLG-EXO in healthy BALB/c mice after intravenous injection of THLG-EXO at a dosage of 20 mg/kg every other day for a week. Compared with PBS group, no deaths and serious body weight loss were observed for the test groups during the study period (data not shown). As is well known, most of the intravenously injected nanoscale lipid vesicles were taken up and eliminated by mononuclear phagocyte system (MPS) [29]. Thus we further investigated the potential pathological lesions induced by exosomes on such organs. Blood biochemistry and hematology analysis were carried out to reveal any potential toxic effect of exosomes on the treated mice. Different biochemistry parameters were tested including the liver function markers such as alanine aminotransferase (ALT), aspartate aminotransferase (AST), and the kidney function markers such as creatinine (CRE) and blood urea nitrogen (BUN). As it could be seen in Table 1, all the above indexes levels remained the same as the PBS treated animals, indicating that THLG-EXO has no obvious hepatic or renal toxicity within the dosage regimen. For the hematological assessment, white blood cell (WBC), red blood cell (RBC) and platelet counts were examined. All the above parameters of THLG-EXO treated group indicated no significant difference compared with the PBS group (Table 1). Furthermore, as shown in Fig. 6 major tissues including heart, liver, spleen, lung and kidney had no obvious histopathological abnormalities or lesions in the THLG-EXO treated group, which suggested that there was no evidence of inflammatory response caused by THLG-EXO. All of these results showed that multiple dosage of THLG-EXO did not cause acute toxicity to the hematological system and major organs in mice.

**Table 1:** Clinical chemistry and hematology parameters for THLG-EXO treated mice

Groups	ALT (IU/L)	AST (IU/L)	BUN (μmol/L)	CRE (μmol/L)	WBC (10^9^/L)	RBC (10^12^/L)	Platelets (10^12^/L)
PBS	55.6 ± 6.4	81.4 ± 12.3	1.52 ± 0.44	14.9 ± 3.1	5.7 ± 1.4	8.2 ± 0.62	1.2 ± 0.15
THLG-EXO	59.6 ± 8.7	78.7 ± 16.3	1.55 ± 0.61	17.5 ± 4.4	5.5 ± 1.1	8.5 ± 0.42	1.1 ± 0.14

*ALT *alanine aminotransferase, *AST* aspartate aminotransferase, *BUN* blood urea nitrogen, *CRE* creatinine, *WBC* white blood cell count, *RBC* red blood cell count

## Discussion

As reported previously, the mechanisms of MDR is very complex and is usually the synergistic result of a combination of several mechanisms. Correcting the lethal alterations in the gene expression by targeting the miRNAs that are known to be responsible for resistance in those cells is potentially a powerful way of reversing the resistance. Recent reports have suggested that miR-21, functioned as an oncogene, is over expressed in most of human malignancies and thus could serve as a diagnostic or prognostic marker for human malignancies [30]. MiR-21 has also been reported to be associated with resistance to chemotherapeutic drugs, including gemcitabine, docetaxel, temozolomide, 5-fluorouracil, doxorubicin and taxol [7, 31,32]. Furthermore, a lot of genes which are involved in chemoresistance, including hMSH2, PTEN, PDCD4 have been experimentally verified to be the target genes of miR-21 [18, 33-35]. Herein, we developed an engineered exosomes to simultaneously deliver miR-21i and chemotherapeutic drug 5-FU to HCT-116^5FR^ cells, to overcome the chemoresistance and thus to increase the effectiveness of the cancer treatment.

Nano-drug-loaded carrier with exquisite modification for binding to cancer cell membranes, the microenvironment, or to cytoplasm, which results in delivery of high drug concentrations to the targeted cancer cells, with reducing toxicity to the other normal cells. An important option which is available for the target specificity is to use ligand based modification on their specific affinity for the membrane protein over-expressed on the tumor cell surface. Her2 is highly expressed in a significant proportion of breast cancer, ovarian cancer and colon cancer cases [36, 37]. The over expression of Her2 is also clearly associated with more aggressive tumor phenotypes and poor prognosis, thus the over expression and accessibility of the extracellular domain of Her2 on tumor cells make it an ideal marker for the receptor mediated drug delivery systems [38, 39]. Although there is no natural ligand, artificial ligands such as antibody, Fab, ScFv, affibody and peptide have been developed for Her2 targeted drug delivery. In this study, in order to endow the target specificity, exosomes were engineered to possess an anti-Her2 affibody on its surface, by fusing the affibody with LAMP2, an abundantly expressed surface protein of exosomes. Affibody molecules are stable, 58 amino-acid Z-domain scaffolds, derived from the IgG binding domains of staphylococcal protein A. Previous studies have reported that anti-Her2 affibody has high sensitivity and specificity for Her2 expression compared with target Her2 peptide, and showed excellent tissue/cell penetration ability. In accordance with these studies, our results also demonstrated that the modification of anti-Her2 affibody to the surface of exosomes achieved cellular tropism of engineered exosomes in vitro and significantly enhanced exosomes accumulation in the tumors in vivo (Fig. 4). However, in the earlier studies, anti-Her2 peptide has often been employed as a targeting ligand for nano-scaled drug delivery systems, such as liposome, nanoparticles, and nano-micelles [40-42]. An important reason behind this is, compared with affibody, peptide has fewer amino acid residues which facilitates the modification of nano-scaled drug delivery systems, but this is not the case with exosomes. As exosomes are naturally derived from cells, they can be easily modified during its biosynthesis.

Furtherly, visualization and tracking of exosomes uptake by target cells in vitro and the pharmacokinetics of exosomes in vivo are technically challenging and often requires extensive sample purification and labeling. PKH, along with other lipophilic dyes such as DiO, DiI, DiR, represents one of the most commonly used dyes to label exosomes. However, previous studies have showed that lipophilic dyes staining of exosomes have many shortcomings while considering at least the following points: (i) they label other lipid-containing entities in the extracellular space; (ii) they tend to form aggregation themselves; (iii) they have different metabolism profile from exosomes in vitro and in vivo [43, 44]. All these problems mentioned above potentially may lead to inaccurate spatiotemporal assessments of exosomes fate. Exosomes surface proteins, at least in part, have been reported to be responsible for the binding and subsequent uptake of exosomes by the recipient cells. Therefore, it is ideal to minimize the perturbation to the exosomes surface proteome during labeling thereby reducing potential disturbance to the exosomes surface protein composition. In this study LAMP2 had been fused at COOH-termini of GFP for exosomes membrane labeling. Western blot analysis demonstrated that the fusion protein tending to be associated with exosomes. Moreover, we successfully observed the engineered exosomes with confocal microscopy and found that the THLG-EXO had a well targeting property to Her2 positive cells in vitro. Yet it is worth pointing out that, the experiment failed when we attempt to detect GFP to study the pharmacokinetics of THLG-EXO in vivo. One possible reason could be, in part, by the nonoptimal spectrum of the green GFP fluorescence (520 nm). At this relatively short wavelength, the emitted radiation is strongly scattered by surrounding tissue. Thus, future studies are needed to seek a proper detectable signaling protein to label exosomes for facilitating the detection in vivo.

While aiming to use endogenous exosomes as nanocarriers for the delivery of exogenous therapeutic cargoes, it is essential to have an efficient drug loading method available. Recently proposed methods described that exogenous drug could be encapsulated in exosomes by electroporation, ultrasound, freeze–thaw cycles [45-47]. Among these methods, electroporation, as the earliest and most widely used, has been shown to be an effective way for the loading of siRNAs, iron oxide and doxorubicin [10, 20, 48]. However, the efficiency of this approach remains a topic of debate. Raemdonckb’s group has found that electroporation can induce strong aggregation of the nucleic acid drug, which might be mistakenly interpreted as encapsulation of the nucleic acid drug into exosomes [49]. In this study, DLS tests showed that the diameter of exosomes slightly increased after electroporation (Fig. 1g), which could be due to an aggregation of a part of nucleic acid. However, in follow-up experiments, the target genes of miR-21 in recipient cells was rescued by THLG-EXO/5-FU/miR-21i, which, at least, suggested that a fairly large number of miR-21i could have been loaded into exosomes by electroporation. These results insisted that during electroporation, aggregation and encapsulation of nucleic acid could occur simultaneously and which of these phenomena dominates, critically might depend on the electroporation buffer and on the electrical parameter. Thus, searching a suitable electroporation solution system and relative technical parameters could be an area of future study.

In colorectal tumors, Nicola Valeri et al. have correlated 5-FU drug resistance to the over expression of miR-21 which directly down-regulates the core MMR proteins hMSH2 and hMSH6 and ultimately leads to a defect in damage-induced G2/M arrest and apoptosis [35]. Naganoet al. have also demonstrated that the miR-21 in HCC cell lines and clinical HCC samples acts as a significant modulator of anti-tumor effect of 5-FU. Transfection of anti-miR-21 rendered HCC cells sensitive to 5-FU, and such sensitivity was weakened by transfection of siRNAs of target molecules, PETN and PDCD4 [35]. In the present study, engineered exosomes were employed to co-deliver 5-FU and miR-21i into HCT-116^5FR^ cells which showed that while co-delivering miR-21i could increase the sensitivity of 5-FU on HCT-116^5FR^ cells by rescuing the expression of hMSH2 and PTEN, and the combination therapy with 5-FU and miR-21i yielded a significantly enhanced antitumor efficacy compared with the single-agent treatment, as reflected in the decrease in tumor cell proliferation in vitro (Fig. 3e), tumor sizes (Fig. 5c) and tumor weights (Fig. 5d) in vivo. Mechanistically, the engineered exosome-based delivery system could efficiently facilitate cellular uptake and significantly down-regulate miR-21 expression in HCT-116 cell lines. Consequently, the down-regulation of miR-21 induced cell cycle arrest, reduced tumor proliferation and increased apoptosis, inhibited the tumor cell migration and suppressed PTEN and hMSH2 expressions, regulatory targets of miR-21. More importantly, the combinational delivery of miR-21i and 5-FU with the same exosomes significantly enhanced the cytotoxicity in 5-FU-resistant colon cancer cells, compared with the single treatment with either miR-21i or 5-FU. Based on these findings, we are confident that 5-FU and miR-21i could be synchronously introduced into colorectal cell to reverse drug resistance by engineering exosomes. The T-Her2-containing exosome developed in this study can thereby be serve as a general approach to deliver and conveniently load miR-21i creating a targeting delivery system, which shown an effectively delivery system for miRNA and chemotherapeutics to HCT-116^5FR^ cells.

## Conclusions

In summary, we have successfully developed a combined strategy that applies the engineered exosomes delivery system to simultaneously deliver miR-21i and chemotherapeutic drug 5-FU to the HCT-116^5FR^ cancer cells for effectively reverse drug resistance and improve the efficiency of cancer treatment. These data clearly indicate that optimization studies regarding the combination of miRNA modulation with chemotherapeutic drugs may contribute to the establishment of a synergistic treatment with the potential to be translated into the clinics in the near future.

## Materials and methods

### Cell culture

5-FU-resistant derivative of the HCT-116 human colon cancer cell line (HCT-116^5FR^) was generated by serial passage of these cells in the presence of increasing 5-FU concentrations as previously described [50]. HEK293T and SGC-7901 cell lines were purchased from the American Type Culture Collection (ATCC) and were routinely maintained in a humidified chamber at 37 °C and 5% CO_2_. HEK293T cells and SGC-7901 cells were grown in Dulbecco modified Eagle medium (DMEM, high glucose), and HCT-116^5FR^ cells in McCoy’s 5A medium, both supplemented with 10% fetal bovine serum and 1% of penicillin/streptomycin (100 units/mL penicillin and 100 μg/mL streptomycin). All cell culture reagents were purchased from Hyclone Laboratories Inc. (Logan, UT, USA). The fetal bovine serum was vesicle-depleted by an overnight ultracentrifugation at 110,000× *g*, followed by filtration through a 0.22 μm Steritop filter (Millipore).

## Vector construction, preparation of lentivirus

Sequence encoding LAMP2 signal peptide, Her2 binding affibody (VDNKFNKEMRNAYWEIALLPNLNNQQKRAFIRSLYDDPSQSANLLAEAKKLNDAQAPK), LAMP2 (segment after the signal peptide) and a flexible peptide linker (GGGGS)_3_ were directly fused into pLVX-GFP-N1 in series by homologous recombination to create target-Her2-LAMP2-GFP (THLG) plasmid. Sequence encoding extracellular region and trans-membrane region of Her2 was directly fused into pLVX-mcherry-N1 to create Her2-mcherry plasmid. Lentivirus was generated by transient transfection of HEK293T cells using psPAX2 and pMD2.G packaging plasmids.

## Isolation of exosomes

The exosomes were purified according to the literature with some modifications [51]. Briefly, the cell medium containing exosomes was harvested by centrifugation at 300×*g* for 5 min to eliminate cells and subsequently centrifuged at 10,000×*g* for 30 min to remove dead cells and cell debris. Finally, the clear supernatant was centrifuged for 90 min at 100,000×*g* to pellet exosomes using an L-100 XP ultracentrifuge (Beckman Coulter, Brea, CA, USA). After discarding the supernatant, the exosomes pellet was resuspended in 1 mL of 1 × PBS to which an additional 25 mL of cold 1 × PBS was added and further ultracentrifuged at 100,000×*g* for 70 min to remove residual media components from the exosomes. At last, exosomes were resuspended into 100 μL of 1 × PBS and stored immediately at − 80 °C. All the centrifugation steps were carried out at 4 °C.

## Exosomes labeling

The fluorescent dye 1, 1′-dioctadecyl-3, 3, 3′, 3′-tetramethylindotricarbocyanine iodide (DiR) was purchased from Biotium (Fremont, CA, USA) and used to label exosomes. Purified exosomes were incubated in the presence of 5 mM DiR for 15 min at 37 ℃, then ultracentrifuged at 120,000×*g*, 90 min to remove the unbounded dye. After being washed twice in PBS with 120,000×*g* centrifugation, the labeled exosomes were resuspended in PBS prior to use.

## Exosomes loading

To load engineered exosomes with exogenous cargoes, miR-21i and 5-FU were transfected by Neon electroporation (1000 V, 10 ms, 2 pulses). Exosomes at a total protein concentration of 10 μg (measured by BCA) were mixed with 400 nm of miR-21i and 10 μg 5-FU in R buffer from the Neon kit (Invitrogen) before electroporation. After electroporation, exosomes were washed two times in PBS with 100,000×*g* centrifugation.

To calculate the cargo loading efficiency, after electroporation treatment, samples were diluted 100 × with PBS and centrifuged at 100,000×*g* for 70 min to remove free miR-21i and 5-FU. Encapsulation of miR-21i in exosomes was analyzed by quantitative reverse transcription PCR (qRT-PCR). RNA was isolated from pellets using TRIzol Reagent according to the manufacturer's recommendations, with minor modifications. On the other hand, spectrophotometer was employed to determine the envelopment rate of 5-FU. EXO/5-FU/miR-21i (50 mg) was distributed in 50 ml of 1 mol/L HCl by sonication for 1 h. The concentration of 5-FU in the supernatant was assayed by UV–Vis spectrophotometer (UV–Vis 8500, Techcomp, China) at 265 nm and the supernatant from vacuous exosomes was used as a contrast. The drug loading capacity of EXO/5-FU/miR-21i was calculated by the following formulas:

$${\text{LC}}\% =\frac{W1}{W2}\,\times100\%$$

where *W*_1_ is the weight of miR-21i or 5-FU encapsulated in the exosomes, *W*_2_ is the gross weight of the EXO/5-FU/miR-21i.

## Qualitative study of Her2^+^ cell binding and uptake of THLG-EXO in vitro

The cellular uptake and distribution of the drug formulations were examined by confocal microscopy and flow cytometry. About 3 × 10^5^ Her2-negative SGC-7901 WT cells and 3 × 10^5^ Her2-mcherry-SGC-7901 cells were seeded in 12-well plates. When the cells reached about 70% confluency, THLG-EXO or LG-EXO was added directly to the cells and incubated for 4 h at 37 °C, respectively. Then the cells were harvested, washed with PBS, and analyzed by flow cytometry (BD Accuri C6 Flow Cytometry) and confocal laser scanning microscopy (Nikon, Japan).

## Assay antitumor activity in vitro

The effect of different exosomes formulations on apoptosis was assessed by using the AnnexinV-FITC kit (Dojindo, Kumamoto, Japan) as per the manufacturer’s protocol. Briefly, HCT-116^5FR^ cells were seeded in the 6-well plates (2 × 10^5^ cells per well) and incubated for 24 h. Free THLG-EXO, THLG-EXO/miR-21i, THLG-EXO/5-FU and THLG-EXO/5-FU/miR-21i were introduced into the cells at a concentration of 150 nM miR-21i and of 10 μg/mL 5-FU, respectively, and incubated further. After 24 h of incubation HCT-116^5FR^ cells were collected, washed twice with cold D-hanks buffer solution, and resuspended in binding buffer (1 × 10^6^ cells/mL). Later 100 μL of HCT-116^5FR^ cells were transferred to a tube, to which 5 μL of FITC-conjugated Annexin V (Annexin V-FITC) and 5 μL of propidium iodide (PI) were added followed by incubated for 15 min at room temperature in the dark. The stained HCT-116^5FR^ cells were further diluted by the binding buffer and directly analyzed by Accuri C6 (BD Biosciences, CA) using CFlow (BD Biosciences, CA) software. The cells were set as positive depending on the fluorescence intensity of Annexin V-FITC or PI. The positive of Annexin V-FITC indicates the out-releasing of phospholipid phosphatidylserine (PS), which happens in the early stage of apoptosis. The positive of PI indicates the damage of cell membrane, which occurs either in the end stage of apoptosis, in necrosis or in dead cells. Therefore, the apoptotic cells were identified as Annexin V-FITC^+^ and PI^−^. The nonviable cells were identified as Annexin V-FITC^+^ and PI^+^ and viable cells as Annexin V-FITC^−^and PI^−^.

The effect of different exosomes formulations on cell cycle was assessed by using the propidium iodide (Beyotime, China). Briefly, the cells were harvested at 24 h after treated by different exosomes formulation and were fixed in 70% ice cold ethanol. The fixed cells were then rehydrated at room temperature for 30 min in PBS buffer containing 2% FCS and 0.1% Tween-20, centrifuged at 1500 g for 10 min and resuspended in 0.5 mL of the above buffer to which RNase A (5 mg/mL) was added. RNase A digestion was carried at 37 ℃ for 30 min, followed by staining with propidium iodide (5 mg/mL). The cells were analyzed using Accuri C6. For calculating the percentage of cells in different phases of the cell cycle, the ModFit software was used.

## Assay tumor targeting and anti-tumor activity in vivo

Six week old female BALB/c nude mice were purchased from Beijing Vital River Laboratories. To establish tumors, mice received subcutaneous flank injections of 1 × 10^6^ HCT-116^5FR^ tumor cells. The tumor targeting study of the exosomes was initiated when the tumor grew to approximately 0.2 cm^3^. To monitor the tumor targeting property of the exosomes for the Her2-positive tumor bearing mice, the in vivo DiR labeled THLG-EXO and LG-EXO were monitored in real-time in live animals using a fluorescence imaging system (Xenogen, Alameda, CA, USA) with the excitation and emission wavelength of 750 and 780 nm respectively. 250 μl of DiR labeled THLG-EXO or LG-EXO was administered via the tail vein injection at a concentration of 1 mg/kg. At 30 min, 1 h and 3 h post-injection, the tumor-bearing mice were anesthetized with pentobarbital and the fluorescence images were captured using the small animal in vivo fluorescence imaging system.

Tumors were established in six-week old female BALB/c nude mice as described above. When tumor volumes reached 50 mm^3^ (volume = 1/2 length × width^2^, measured with a vernier caliper), mice were randomly divided into four groups, with six mice per treatment group, as follows: Group 1: THLG-EXO/5-FU /miR-21i; Group 2: THLG-EXO/5-FU; Group 3: THLG-EXO/miR-21i; group 4: THLG-EXO control. Intravenous injections at 2 mg exosomes per mouse were performed through tail vein, for three days a week (Monday, Wednesday and Friday). The tumor volume was monitored by bioluminescent imaging once in a week using an in vivo IVIS 100 bioluminescence/optical imaging system (Xenogen, Alameda, CA, USA). D-Luciferin (Xenogen) dissolved in PBS was injected i.p. at a dose of 150 mg/kg, 10 min before measuring the light emission. General anesthesia was induced with 5% isoflurane and continued during the procedure with 2.5% isoflurane introduced via a nose cone. After acquiring photographic images of each mouse, luminescent images were acquired with various exposure times. The resulting gray scale photographic and pseudo-color luminescent images were automatically superimposed by the IVIS Living Image software to facilitate matching the observed luciferase signal with its location on the mouse. Regions of interest (ROI) were manually drawn around the bodies of the mice to assess signal intensity emitted. Luminescent signal was expressed as photons per second emitted within the given ROI. All procedures were approved by the Committee on the Ethics of Animal Experiments of the Health Science Center of Southeast University (Nanjing, China).

For TUNEL analysis, in situ cell apoptosis detection kit (BOSTER, Wuhan, China) was used to determine apoptosis in tumor sections. According to the manufacturer's instructions, the tumor slides were incubated with TdT and DIG-d-UTP including binding buffer followed by incubation 2 h at 37 ℃. Then 50 μl blocking solution was added at the slides, 30 min at room temperature, and followed by biotinylated anti-digoxigenin antibody and SABC reagents incubation. The staining processes were performed with diaminobenzidine (DAB, BOSTER, Wuhan, China) colorimetric reagent solution and hematoxylin. Those with brown-yellow particles in the nucleus are positive cells, which are considered to be apoptotic cells.

### Western blot analysis

Total exosome or cell protein was prepared by resuspending the samples in RIPA lysis buffer (Beyotime, China) containing 1 mM phenlymethylsulfonyl fluoride, 5 μg/mL leupeptin, 2 μg/mL aprotinin, and 1 μg/mL pepstatin A. About 30–40 μg of protein from each sample was separated on a 10% SDS-PAGE gel, transferred to a PVDF membrane, and blotted with antibodies against CD63, CD9, CD81(Santa Cruz, CA, USA), GFP (Sigma-Aldrich, St Louis, MO, USA), hMSH2 (Santa Cruz, CA, USA), PTEN (Santa Cruz Biotechnology, Santa Cruz, CA) or GAPDH (Sigma-Aldrich, St. Louis, MO, USA). Chemiluminescence was detected using the ECL detection solution (cwbiotechnology, Beijing, China).

## RNA isolation and quantitative real-time reverse transcription-PCRs.

RNA was isolated from cells using RNAiso Plus reagent (TaKaRa, Dalian, China) according to the manufacturer’s instructions. MiRNA levels were quantified using TaqMan miRNA assays (Applied Biosystems, Carlsbad, CA). Copy numbers were calculated based on a standard curve created using synthetic RNA. MiRNA levels were normalized by the U6 levels. Quantitative PCRs was performed using ABI7500 Real-Time PCR System (Applied Biosystems).

## In vivo safety evaluation

Eight female BALB/c mice were randomly divided into two groups. One group received an intravenous injection of THLG-EXO (20 mg/kg) at one dose every other for a week and the other group was treated with PBS as control. Blood samples and major organ tissues were collected at 24 h after the last administration, for hematologic and histochemistry analysis. White blood cell (WBC), red blood cell (RBC) and platelet (PLT) were measured by Advia 120 Automated Hematology Analyzer (Bayer Ltd., Germany). The serum aspartate transaminase (AST), alanine transaminase (ALT), urea nitrogen (BUN) and creatinine levels were analyzed by Hitachi 7080 Chemistry Analyzer (Hitachi Ltd., Japan). Major organs such as heart, lung, liver, spleen, and kidney were fixed with paraformaldehyde for 48 h and embedded in paraffin. Each section was cut into 5 mm, processed for routine hematoxylin and eosin (H&E) staining, and visualized under a Leica microscope.

### Statistical analysis

Data are presented as the mean ± standard deviation. One-way analysis of variance was used to determine significance among groups, after which post-hoctests with the Bonferroni correction were used for comparison between individual groups. A value of p < 0.05 was considered to be significant.

## Data Availability

All data generated or analyzed during this research are included in this manuscript.

## Abbreviations

5-FU: 5-fluorouracil
CRC: colorectal cancer
MDR: multidrug resistance
miR-21i: miR-21 inhibitor oligonucleotide
LAMP-2: lysosome-associated membrane proteins 2
Her2: human epidermal growth factor receptor 2
MVB: multivesicular bodies
HCT-116^5FR^: 5-fU-resistant derivative of the HCT-116 human colon cancer cell line
THLG: target-Her2-LAMP2-GFP
LG: lAMP2-GFP
DiR: 1,1′-dioctadecyl-3, 3, 3′, 3′-tetramethylindotricarbocyanine iodide
qRT-PCR: quantitative reverse transcription PCR
WBC: white blood cell
RBC: red blood cell
PLT: platelet
AST: aspartate transaminase
ALT: alanine transaminase
BUN: urea nitrogen
H&E: hematoxylin and eosin
TUNEL: tdT dUTP nick end-labeling
